# Positive Mental Health Scale (PMHS) in Parents of Children with Cancer: A Psychometric Evaluation Using Item Response Theory

**DOI:** 10.3390/cancers15102744

**Published:** 2023-05-13

**Authors:** Filiberto Toledano-Toledano, Said Jiménez, José Moral de la Rubia, Cesar Merino-Soto, Leonor Rivera-Rivera

**Affiliations:** 1 Unidad de Investigación en Medicina Basada en Evidencias, Hospital Infantil de México Federico Gómez, National Institute of Health, Dr. Márquez 162, Doctores, Cuauhtémoc, Mexico City 06720, Mexico; 2 Unidad de Investigación Multidisciplinaria en Salud, Instituto Nacional de Rehabilitación Luis Guillermo Ibarra Ibarra, Calzada México-Xochimilco 289, Arenal de Guadalupe, Tlalpan, Mexico City 14389, Mexico; 3 Dirección de Investigación y Diseminación del Conocimiento, Instituto Nacional de Ciencias e Innovación para la Formación de Comunidad Científica, INDEHUS, Periférico Sur 4860, Arenal de Guadalupe, Tlalpan, Mexico City 14389, Mexico; 4 Facultad de Psicología, Universidad Autónoma de Nuevo León, Dr. Carlos Canseco, 110, Esq. Dr. Aguirre Pequeño, Col. Mitras Centro, Monterrey 64460, Mexico; 5 Instituto de Investigación de Psicología, Universidad de San Martín de Porres, 34, Lima 15011, Peru; sikayax@yahoo.com.ar; 6 Centro de Investigación en Salud Poblacional, Instituto Nacional de Salud Pública, Av. Universidad No. 655 Col. Santa María Ahuacatitlán, Cuernavaca 62100, Mexico; lrivera@insp.mx

**Keywords:** mental health, parents, psychometric evaluation, validity, IRT, PMHS, children with cancer, quality of life, positive mental health scale, family caregivers

## Abstract

**Simple Summary:**

The Positive Mental Health Scale (PMHS) is the most widely used instrument to measure positive mental health worldwide. However, there is no theoretical, clinical, or empirical knowledge regarding the psychometric properties of the PMHS in parents of children with cancer, indicating a significant gap in the literature. In this study, we aimed to investigate the psychometric properties of the PMHS in parents of children with cancer using a psychometric evaluation based on item response theory (IRT). To accomplish this purpose, an ex post facto study with a cross-sectional design and non-probability sampling was conducted to evaluate 623 parents of children with cancer at the National Institute of Health in Mexico City. The main findings indicate that the IRT graded response model validates the single latent trait model. The PMHS demonstrated adequate reliability and was found to be a valid, reliable, and culturally relevant instrument that can be used in parents of children with cancer.

**Abstract:**

Mental health is currently a public health issue worldwide. However, evidence is lacking regarding the validity of the instruments used to measure and assess positive mental health in specific populations. The objective of this study was to evaluate the psychometric properties of the PMHS using IRT. A cross-sectional retrospective study with non-probabilistic convenience sampling was conducted with 623 parents of children undergoing cancer treatment at the National Institute of Health in Mexico City. The participants responded to a battery of tests, including a sociodemographic questionnaire, the PMHS, Measurement Scale of Resilience, Beck Depression Inventory, Inventory of Quality of Life, Beck Anxiety Inventory, an interview regarding caregiver burden, and the World Health Organization Well-Being Index. PMHS responses were analyzed using Samejima’s graded response model. The PMHS findings indicated that the IRT-based graded response model validated the single latent trait model. The scale scores were independent of depression, anxiety, well-being, caregiver burden, quality of life, and resilience. The PMHS scores were associated with low subjective well-being. The PMHS findings reveal that from an IRT-based perspective, this scale is unidimensional and is a valid, reliable, and culturally relevant instrument for assessing positive mental health in parents of children with chronic diseases.

## 1. Introduction

Parents caring for their children with cancer experience individual, familial, and sociocultural challenges [1,2,3,4]. In addition, caregiving impacts the quality of life, caregiver profile and mental health of families caring for children with cancer [5,6]. The concept of positive mental health was developed by Jahoda [7] and has been studied from individual and psychological perspectives, both as a personality trait and as a state that arises from social situations. This author proposes that mental health is a matter of individual character and that although it may be influenced by social contexts, the condition of health or illness corresponds to individuals. For this author, mental health is a human value that can vary by culture and for which there is no single definition; however, some domains that make up positive mental health have been identified: attitudes toward oneself, growth and self-actualization, integration, autonomy, perception of reality, and mastery of the environment [7].

The World Health Organization (WHO) has defined mental health as a state of well-being in which the person manifests his or her capacities to cope with the daily stress of life, to work productively and to contribute to the development of their community [8]. In this context, the results of a systematic review of the literature suggest that positive mental health is the absence of disorders, or the presence of certain personality traits that protect the individual from becoming ill or contribute to their recovery or rehabilitation from difficulties or disorders [9]. In the Mexican population, positive mental health has been defined as a set of personality characteristics and biopsychosocial skills that a person has to achieve vital goals, self-realization, states of well-being and adaptation [10].

Based on the studies of Jahoda in 1954, Canut developed the first scale of positive mental health. Canut proposed reducing Jahoda’s model to a simpler structure of six factors (personal satisfaction, prosocial attitude, self-control, autonomy, problem solving and self-actualization, and interpersonal relationship skills). The final scale had 39 items, and the internal consistency as measured by alpha was α = 0.89 [11,12].

From Lluch’s pioneering studies, other scales have been developed to assess positive mental health: the Positive Mental Health Questionnaire (PMHQ), which evaluates six factors and comprises 39 items [13]; the Mental Health Continuum–Short Form (MHC-SF) scale, which evaluates three factors, namely, emotional, psychological and social well-being [14]; the 12-item General Health Questionnaire (GHQ-12) [15]; the Positive Mental Health Instrument (PMHI), which assesses six factors, namely, general coping, personal growth and autonomy, spirituality, interpersonal skills, emotional support and global affect, and consists of 47 items [16]; the Achutha Menon Center Positive Mental Health Scale (AMCPMHS), which evaluates four dimensions and consists of 20 items [17]; the 19-item Positive Mental Health (PMH-19) scale [18]; the Positive Mental Health (PMH) Scale, which is a short, one-dimensional scale that consists of nine items rated on Likert scales [19]; and the Rapid Positive Mental Health Instrument (RPM), a six-item unidimensional scale [20]. For the Mexican population, the Mental Health Scale for Adults was developed, consisting of seven factors, namely, emotional cognitive well-being, the environment, social skills, empathy and social sensitivity, physical well-being, self-reflection and psychological discomfort; these factors are used to group 83 items, and the internal consistency was reported to be α = 0.962, explaining 39.12% of the variance [10]. Therefore, the empirical evidence available suggests that the scales designed to measure positive mental health have indicators based on constructs that include resilience, self-esteem, self-efficacy, optimism, satisfaction with life, hope, perceptions and judgments about the sense of coherence and meaning of life and social integration [21].

The PMHS was originally developed in the German population. It is a short, one-dimensional self-report scale (assessing positive emotionality related to positive mental health) and is person-centered. It consists of nine items scored on a Likert scale ranging from 1 (never) to 5 (always). Higher scores indicate more positive mental health [19]. The results showed that the scale is unidimensional and invariant between scores and between samples, and the correlation over time was >0.40, with high internal consistency (α = 0.93), good retest reliability (r > 0.74), and good construct validity (convergent and discriminant), as well as sensitivity to therapeutic change (Cohen’s d = 0.50). It has been helpful in evaluating positive mental health in the community and mental health care.

The items of the PMHS were derived from the Trierer Personality Inventory [22], the Freiburg Personality Inventory, the Mental Health Scale, the Berner Well-Being Inventory [23] and an item formulated by Lukat et al. [19]. Lluch’s Positive Mental Health Scale has been used in the adult population [24]. It was validated in Mexican children [25]. Using this scale, it was shown that among a group of Mexican adolescents, 38.3% exhibited high levels of positive mental health and 16.1% exhibited very high levels of positive mental health, dominated by the dimension of personal satisfaction [26].

The measurement and evaluation of mental health have been conducted in various contexts and in people with chronic physical health problems. Most participants had medium or high levels of positive mental health, and the variables that negatively affected them were old age, the doses and consumption of multiple medications and the frequent consumption of painkillers. The type of health problem was not found to influence the levels of positive mental health [27]. Positive mental health was shown to moderate the impact of depression on suicidal ideation, such that at higher levels of positive mental health, the severity of depression was not associated with suicidal ideation over time [28]. Likewise, suicidal ideation was less likely when positive mental health translated into a positive affect [29]. In addition, the absence of positive mental health in adults (regardless of age, sex, race, or education level) has been shown to increase the likelihood of mortality from all causes (smoking, physical inactivity, cardiovascular disease, and HIV/AIDS) [30]. A systematic review that evaluated positive mental health programs found that these programs improved the mental health of adults [31].

Most studies that have assessed positive mental health in parents of children with chronic diseases have found a higher incidence of anxiety among them [32]. For example, a study reported that the mental health of caregivers of children with diabetes is affected by the age of the caregiver, changes in health and kinship. Substantial stress experienced by the primary caregiver, leading to anxiety and depression, can seriously affect the recovery rate of stroke patients, resulting in a higher risk of mortality [33]. Studies focused on parents of children with cancer have evaluated mental health, but not positive mental health, and have shown that parents of patients with lung cancer experience negative effects on their physical and mental health. Furthermore, the mental health of caregivers was strongly associated with major life events [34]. Parents of cancer patients experience mental fatigue, expressed as trouble concentrating, trouble remembering things, and irritability [35]. The mental health of primary caregivers of cancer patients predicts future caregiver burden and family functioning [36].

There is scientific evidence of the validity and reliability of the PMHS in various contexts, such as populations and cultures, using classical test theory (CTT); however, no studies have reported empirical findings on the psychometric properties of the PMHS analyzed with item response theory (IRT) in parents of children with cancer, although the IRT offers detailed information about both the items and the precision in the measurement of the construct [37,38].

Thus, to address these knowledge gaps, the aim of the present study was to analyze the psychometric properties of the PMHS. To this end, the present study aimed to evaluate the psychometric properties of the PMHS using IRT in parents of children with cancer. To achieve this aim, we had the following five objectives: (1) to fit and compare two IRT models (the graded response model (GRM) and generalized partial credit model (GPCM)) for PMHS ordinal responses in parents of children with cancer; (2) to obtain the parameters of discrimination and difficulty in constructing the characteristic curves of the PMHS items; (3) to calculate the information functions of each item and the test and estimate the expected PMHS scores from the PMHS scores in the latent trait; (4) to estimate the latent trait scores of parents and relate them to the observed PMHS scores in parents of children with cancer; and (5) to determine the convergent and divergent validity of the PMHS.

## 2. Materials and Methods

### 2.1. Participants

A total of 623 parents of children hospitalized with cancer were interviewed at the Hospital Infantil de México Federico Gómez National Institute of Health in Mexico City. An observational and cross-sectional study was conducted using a nonprobabilistic convenience sampling technique. The inclusion criteria for the study were as follows: (1) being a family caregiver of a child receiving cancer treatment, (2) being at least 18 years old, and (3) signing the informed consent form. The exclusion criteria were as follows: (1) inability to read and write or (2) refusal to participate in the study. The deletion criteria included partial or incomplete responses to the psychosocial measurement instruments.

### 2.2. Measuring Instruments

The Sociodemographic variables questionnaire (Q-SV) for family caregivers of children with chronic diseases [39] contains 20 items and was used to collect sociodemographic, medical, sociocultural and family data from families of children with chronic diseases.

Positive Mental Health Scale (PMHS) [19]: The present study used the short version revised by Lukat et al. [19], which is composed of 9 items rated on a 5-point Likert scale ranging from 0 (never) to 4 (always). A higher score represents higher levels of positive mental health. The scale assesses positive aspects of health and life experiences (e.g., “I am often carefree and in good spirits”, “I enjoy my life”, “I manage well to fulfill my needs”, “I am in good physical and emotional condition”).

Measurement Scale of Resilience (RESI-M) [40]: This scale has been validated in family caregivers of children with cancer [41]. This scale contains 43 items rated on a four-point Likert scale, ranging from 1 “strongly disagree” to 4 “strongly agree”, and measures overall resilience and resilience in five dimensions: strength and self-confidence (19 items), social competence (eight items), family support (six items), social support (five items) and structure (five items). Among 330 family caregivers, the overall internal consistency of the 43 items of the RESI-M was excellent (α = 0.95, ω = 0.96). A unidimensional confirmatory factor analysis (CFA) in the present sample obtained the following fit indices: X^2^ = 8197.2, df = 860, *p* value < 0.001, CFI = 0.55, TLI = 0.53, RMSEA = 0.12, SRMR = 0.10.

Beck Depression Inventory II (BDI-II) [42]: This inventory has been validated for family caregivers of children with chronic diseases [43] and includes 21 items, each with four statements that assess depressive symptomatology and episodes. BDI-II uses a rating scale from 0 to 3. The overall internal consistency of the 21 items was excellent (α = 0.90, ω = 0.91). A unidimensional CFA analysis in the present sample obtained the following fit indices: X^2^ = 904.7, df = 189, *p* value < 0.001, CFI = 0.83, TLI = 0.82, RMSEA = 0.08, SRMR = 0.06.

Beck Anxiety Inventory (BAI; [44]): This scale has been validated in family caregivers of children with cancer by Toledano-Toledano et al. [45]. With 16 items, this inventory assesses anxious symptomatology using a four-point scale, ranging from 0 “Little or nothing” to 3 “Severely”. In the present sample, the overall internal consistency of the 21 items was excellent (α = 0.92, ω = 0.95). A unidimensional CFA analysis in the present sample obtained the following fit indices: X^2^ = 1343.5, df = 170, *p* value < 0.001, CFI = 0.84, TLI = 0.82, RMSEA = 0.10, SRMR = 0.06.

WHO Quality of Life Assessment (WHOQOL-BREF) [46]: This assessment has been validated in a Mexican population [47]. This inventory includes 26 items rated on a five-point Likert scale ranging from 1 to 5. Two items are general questions about quality of life, and the remaining items are grouped into the following dimensions: seven items for physical health (α = 0.72, ω = 0.83), six items for psychological health (α = 0.63, ω = 0.67), three items for social relations (α = 0.65, ω = 0.69) and eight items for environment (α = 0.76, ω = 0.83). The overall internal consistency of the 26 items was excellent (α = 0.90, ω = 0.92). A CFA analysis with four first-order factors and one second-order factor using the present sample obtained poor fit indices: X^2^ = 1948.4, df = 226, *p* value < 0.001, CFI = 0.66, TLI = 0.62, RMSEA = 0.11, SRMR = 0.09.

Zarit Burden Interview (ZBI) [48]: This interview, which has been validated in a Mexican population [49], assesses the subjective burden, attitudes and emotional reactions of the caregiver when faced with the responsibility of care and the perception of the situation. In the present study, only the ZBI total score was used, and its overall internal consistency was excellent (α = 0.90, ω = 0.90). A unidimensional CFA analysis in the present sample obtained the following fit indices: X^2^ = 1863.5, df = 209, *p* value < 0.001, CFI = 0.64, TLI = 0.60, RMSEA = 0.11, SRMR = 0.09.

World Health Organization Well-Being Index (WHO-WBI; [50]): It consists of two factors that explain 62.53% of the total variance: the first factor contains five items related to psychological well-being (α = 0.83), and the second factor contains four items related to physical well-being (α = 0.81). The overall internal consistency of its 9 items was good (α = 0.89, ω = 0.92). A unidimensional CFA analysis in the present sample obtained the following fit indices: X^2^ = 311.8, df = 35, *p* value < 0.001, CFI = 0.90, TLI = 0.88, RMSEA = 0.11, SRMR = 0.05.

### 2.3. Procedure

The specific process used for the cross-cultural adaptation of the PMHS instrument was based on the back-translation method [51]. Initially, the instrument was translated from English into Spanish. Then, the Spanish translation was back-translated into English by independent translators who did not know the objectives of the study. Subsequently, a group of specialists reviewed the disagreements between both translations. These discrepancies were resolved by considering the semantic content of the original PMHS and the cultural context of the Spanish language in Mexico. Finally, the PMHS version translated into Spanish was administered to 80 parents who were voluntarily recruited at the Hospital Infantil de México Federico Gomez, National Institute of Health. These parents were asked about the clarity and understandability of the items, and the items causing confusion were modified. The parents evaluated the items on a three-point scale, and free responses were allowed such that the participants could explain the confusing or unclear aspects of each item.

The data collection was performed by trained personnel at the Evidence-Based Medicine Research Unit of the National Institute of Health under the direction of the first author of this study. The data collection process lasted approximately five months. The parents were evaluated by trained personnel and the corresponding author, who verified the inclusion and exclusion criteria during an interview, which was conducted in the wards of the Hematology-Oncology Service of the Hospital Infantil de México Federico Gómez, National Institute of Health.

All the parents interviewed were invited to participate voluntarily; the objectives of the research were explained to them, and all of their concerns regarding the study were addressed. The parents who agreed to participate signed informed consent forms and answered the instruments individually during a single session. Participants did not face any consequences for withdrawing their consent, as specified on the informed consent sheet. Before collecting the completed instruments, the interviewer checked that there were no questions without answers. If there were questions without answers, the participant was asked to respond to them. In this manner, we managed to prevent missing values.

### 2.4. Ethical Considerations

This study is a part of a research project (HIM/2015/017/SSA.1207, “Effects of mindfulness training on psychological distress and quality of life of the family caregiver”) approved by the Research, Ethics, and Biosafety Commissions of the Hospital Infantil de México Federico Gómez National Institute of Health in Mexico City. While conducting this study, the ethical rules and considerations for research with human subjects currently enforced in Mexico [52] and those outlined by the American Psychological Association [53] were followed. All parents were informed of the objectives and scope of the research and their rights according to the Declaration of Helsinki [54]. The parents who agreed to participate in the study signed an informed consent form. Participation in this study was voluntary and did not involve payment.

### 2.5. Item Response Theory Modeling

Due to the ordinal nature of the PMHS data, two item response theory (IRT) models for polytomous items were fitted and compared: a generalized partial credit model (GPCM) [55] and a graded response model (GRM) [56]. Both the GPCM and the GRM assume that the response categories of items are ordinal, with items higher on the hierarchy reflecting higher levels of the evaluated latent trait. For each item, the two models include a “discrimination” parameter or slope (a), as well as k−1 “difficulty” parameters or thresholds ($b_{1},b_{2},b_{3},b_{4}$), in which k is the number of response options. The parameter a of each item allows its discrimination to be identified; items with higher a values distinguish better among individuals with low or high levels of the latent trait, while the difficulty parameters ($b_{1},b_{2},b_{3},b_{4}$) allow us to model the probability of choosing the different response options for an item. According to Baker [57], the minimum discriminative threshold among respondents for a parameters is 0.65, and estimates higher than a>1.34 are classified as providing “high” discrimination. In the case of the GPCM, the thresholds corresponded to the level of the latent trait necessary to move from one adjacent category to another, while in the GRM, the parameter $b_{k}$ indicates the “difficulty” of the category in question.

Since the two models can be fitted for responses to the PMHS, their goodness of fit was compared using the likelihood ratio test, χ^2^ test, and different information criteria. After the models were compared to identify the best fitting model, the item characteristic curves (ICCs) were examined. The ICC represents the probability of choosing the different response options depending on a range of values in the latent trait (θ). Subsequently, the item and test information functions were obtained, as well as the expected total scores based on the levels of −6≤θ≤6 in the latent trait. Additionally, the latent trait scores of each caregiver were estimated, and the relationship with the observed total scores was evaluated. To investigate the validity of the PMHS latent scores, Pearson and Spearman correlation analyses were performed to determine the relationships of the latent scores with the sum of the observed scores for resilience (RESI-M), depression (BDI-II), anxiety (BAI), caregiver burden (ZBI), quality of life (WHO-QOL) and global well-being (WHO-WBI). A positive correlation (convergent validity) was expected for resilience, quality of life, and well-being; similarly, a negative correlation (divergent validity) was expected for depression, anxiety, and caregiver burden. All analyses and visualizations were performed using the R packages *mirt* 1.37.1 [58] and *ggplot2* 3.4.1 [59].

## 3. Results

### 3.1. Characteristics of the Parents

The sample included 507 women (81.4%) and 116 men (18.6%) aged 18 to 49 years, with an average age of 31.6 years (SD = 7.5). The median and mode of the number of children was two, and the range was from 0 to 10. More details are provided in Table 1.

### 3.2. Model Comparisons

Both the GPCM and GRM yielded very similar goodness-of-fit indices, where usually accepted values are RMSEA ≤ 0.06, SRMSR ≤ 0.08, CFI ≥ 0.96, TLI ≥ 0.90 [60]; the GPCM yielded RMSEA = 0.10, SRMR = 0.07, TLI = 0.95 and CFI = 0.96, while the GRM yielded RMSEA = 0.10, SRMR = 0.07, TLI = 0.94 and CFI = 0.96. However, the models differed in the *M_2_* statistic (M_2_* = 222.14 for the GRM, while M_2_* = 208.07 for the GPCM), as well as in all the information criteria (Table 2). The goodness-of-fit indices shown in Table 2 do not have a specific scale; instead, they reflect differences between the observed covariance matrix and the model-predicted covariance matrix. Thus, the less discrepancy there is between the observed matrix and the one predicted by the model, the better the fit. Therefore, the interpretation of the indices involves determining which of the models had the lowest value of the index, which reflects a better fit [61]. These indicators suggest that the GRM was superior to the GPCM; thus, the GRM was considered the best model for the investigated data.

### 3.3. Parameter Estimation and Item Characteristic Curves

**Figure 1:**
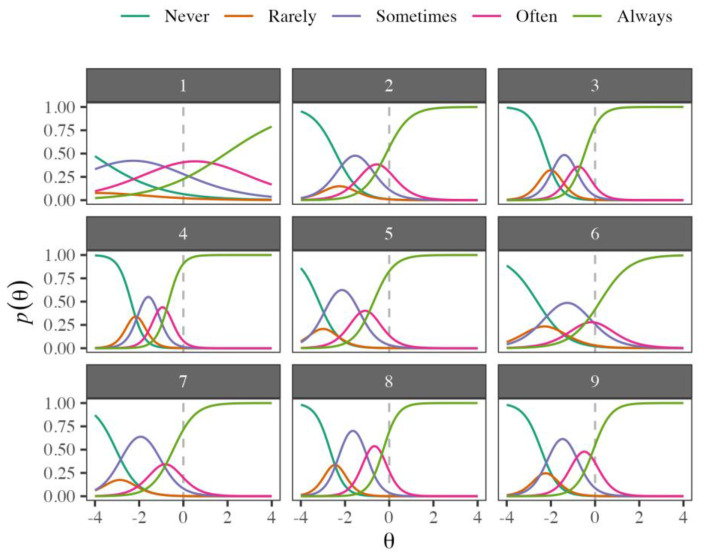
Item characteristic curves of the PMHS. Probability of selecting the different response categories for each item depending on levels of the latent trait.

As the GRM was superior to the GPCM in terms of fit, only the information corresponding to the former model is reported. The item-level parameters are shown in Table 3. Except for the first item, the a parameters were higher than 0.65 (proposed by Baker [57] as the minimum discrimination threshold between respondents), and the other items had higher estimates of a>1.34, which is classified as high discrimination. The item with the highest discrimination was number 4 (“In general, I am confident”), and the item with the worst discrimination was item 1 (“I am often carefree and in good spirits”). The effect of the a parameter can be seen in the slopes of the ICCs in Figure 1; items with higher a scores had steeper slopes, particularly for the *Always* option.

The ICCs depicted in the panels of Figure 1 display how the probability of choosing a category changes depending on the score in the latent trait θ. Functions that correspond to the five response options are shown in different colors, and the dotted lines indicate the average score in the latent trait. Except for item 1, the most likely response category for parents with a latent score at the average value (θ=0) is the option *Always*. This indicates that it is relatively “easy” to select the highest response category or that it is not necessary to have very positive mental health to choose it. An inspection of Figure 1 revealed that for virtually all items, the most likely response options for the broadest range of θ values are the categories *Never*, *Sometimes*, and *Always*. The probability of selecting *Rarely* never exceeded the probability of the other options for any value of θ. Likewise, the category *Often* was more likely in only very restricted regions of θ. For items 1, 2, 3, 4, 6, 8, and 9, this indicates that the instrument may not require inclusion of the option *Rarely*.

Table 3 also shows the threshold estimates ($b_{1},b_{2},b_{3},b_{4}$), which are frequently interpreted as the “difficulty” parameters. In the GRM, thresholds are ordered from lowest to highest and indicate the score in the latent trait θ necessary to select a specific category or a higher option. For example, item 2 (“I enjoy my life”) has $b_{1}=-2.41$, which indicates that individuals with θ≥−2.41 would choose at least the *Rarely* option. Likewise, the same item has $b_{4}=-0.14$, which means that for an individual to select *Always* on this item, she needs a score in the latent trait of θ≥−0.14. As the latent trait scores increase (e.g., indicating greater positive mental health), the probability of selecting the higher-ranking categories also increases.

### 3.4. Item and Test Information Functions and Expected Total Scores

**Figure 2:**
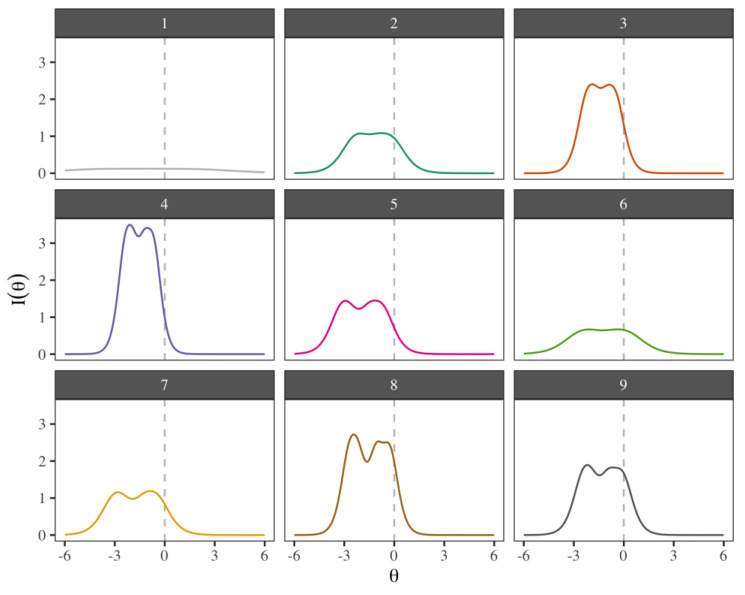
Item information functions of the PMHS. The information provided by each item is shown for latent trait scores in the range of −6≤θ≤6. More information reflects lower standard errors.

Test and item information are measures of the latent trait estimate precision; more information indicates a lower standard error and vice versa. In Figure 2, the item information functions are shown, depending on a range of scores in the latent trait of −6≤θ≤6. Each panel shows the θ values for which the item yields more information. Most of the items provide more information for θ<0 (scores in the latent trait lower than the average). Notably, the items that provide more information on positive mental health are number 4 (“In general, I am confident”), 8 (“Much of what I do brings me joy”), and 3 (“All in all, I am satisfied with my life”), while the item that provided the least information on positive mental health was number 1 (“I am often carefree and in good spirits”).

**Figure 3:**
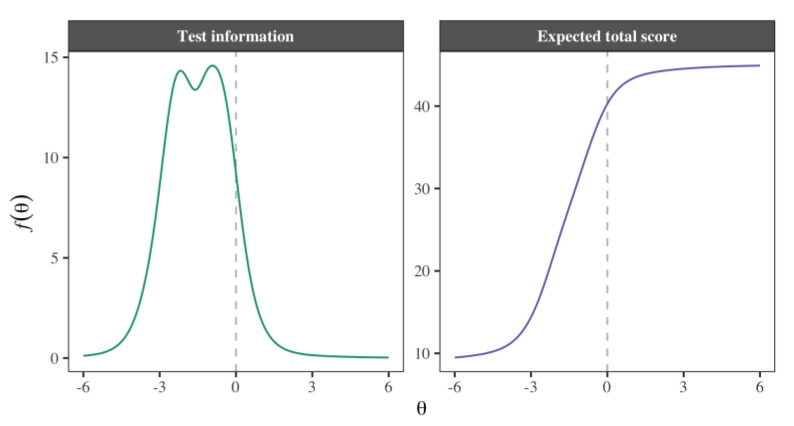
Test information function and expected total score on the PMHS. Left, the sum of the item information, which makes up the test information function. Right, the expected total score based on a range of values −6≤θ≤6 in the trait.

The sum of the item information yields the entire test information, as shown on the left side of Figure 3. The information function indicates regions of the latent trait that the instrument measures with greater precision; in this case, greater information was obtained for latent trait scores of −3≤θ≤0.9, which indicates that the instrument produces more information on the mental health of individuals with low to moderate levels of the trait. The expected total score curve, shown on the right side of Figure 3, displays the expected score according to the value of the latent trait. Intuitively, in the range of −6≤θ≤0, the function indicates that lower levels of θ are related to lower test scores, while higher levels predict higher scores. However, when θ≈0, the change rate begins to decrease until it practically stops when the levels of the trait are slightly above average. This illustrates that the instrument provides substantial information for low to moderate levels of the latent trait and very little information for high levels.

### 3.5. Latent Trait Estimates

**Figure 4:**
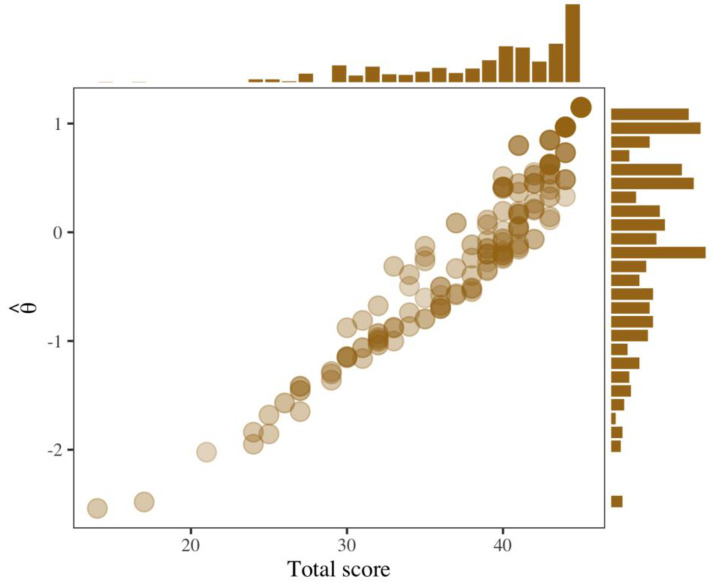
Relationship between latent trait estimates and observed total scores on the PMHS. The points represent the position of a caregiver in the two dimensions, and the histograms show the marginal distributions of the observed test score and the latent estimates.

Finally, the latent trait estimation in the 633 parents yielded an average value (and a standard deviation) of −0.07 (0.85). The relationship between the estimates of the trait and test total score, obtained from the sum of the scores associated with each response category, is shown in Figure 4. This figure presents the rank of each caregiver in both dimensions (according to point values); it also includes histograms that represent the marginal distribution of each variable. Both distributions have a negative bias; however, in the latent estimates, there is a cluster of parents with scores slightly below zero; thus, despite the bias of the distribution, more than 50% of the individuals had negative measurements. Although a positive correlation can be observed between the latent and observed scores, it is worth noting that the same total score predicts different values of $\hat{\theta }$; although all items are scored in the same way (i.e., all are worth a maximum of five points), the amount of positive mental health information each provides is different. The items that provide more information contribute differentially to the estimates of $\hat{\theta }$.

### 3.6. Concurrent Validity

**Figure 5:**
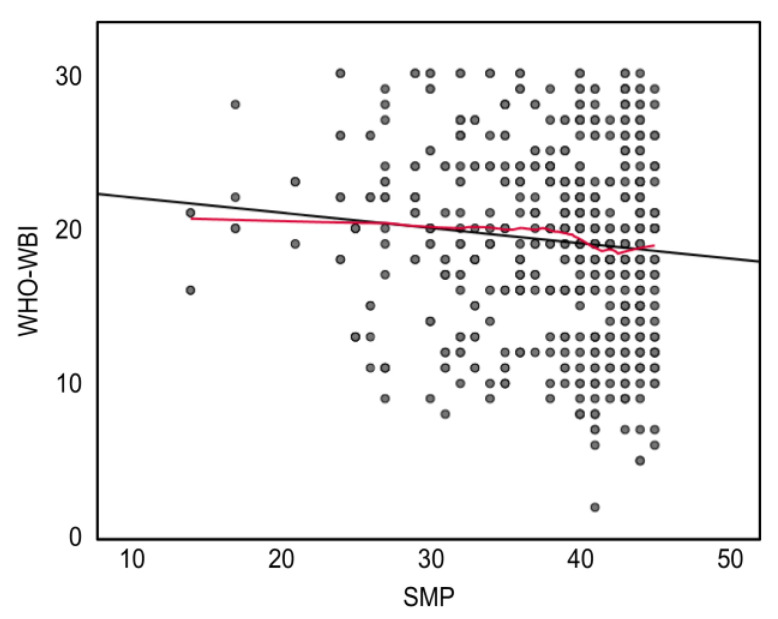
Linear (black line) and loess-kernel (red line) fit between the PMHS and WHO-WBI scores.

It is essential to mention that the scales used in this section did not show adequate fit indices in the one-dimensional confirmatory factor analyses performed on the present sample. For this reason, these results and the interpretations derived from this analysis should be taken with caution. The strength of the linear correlations (Pearson’s correlation coefficient) between the PMHS score and the external constructs was close to zero. The monotonic association, as assessed by Spearman’s correlation analysis, was also very similar to the linear correlations (Table 4). The association between PMHS and WHO-WBI scores was negative; this counterintuitive result was inspected visually using linear and loess-kernel fit lines. Figure 5 shows the effect of possible outliers (low PMHS scores and medium WHO-WBI scores). This effect can be inferred from the difference in this bivariate region, where the loess-kernel adjustment (red line) does not show a decrease in the linear adjustment (black line). Thus, the estimated association between these scores may be closer to zero.

## 4. Discussion

The main objective of the study was to evaluate the measurement properties of the PMHS using a modern psychometrics approach: IRT. This analytic approach is novel because it represents the first evaluation of the PMHS, applying the IRT graduated response and partial credit models. The first aim was to fit and compare the two IRT models mentioned above for ordinal data from parents of children with cancer. The GRM was more appropriate for the data than the GPCM since it had a better fit. Furthermore, a unidimensional scale was obtained, consistent with previous findings using other analysis techniques, such as confirmatory factor analysis (CFA), through maximum likelihood estimation from the item covariance matrix [19] and a model with unconstrained factor loadings and intercepts [62].

Regarding the second aim, the discrimination and difficulty parameters of the nine items of the PMHS were different, which supports the idea that all the items contribute differentially to the dimensions of positive mental health. This is not a problem for the measurement of attributes; indeed, it is a realistic expression of the differential content of the items and the conceptual structure of the measured construct. The fact that all the items presented high or very high discrimination indicates that they have the ability to distinguish with great accuracy between individuals with low or high levels of the dimension evaluated in the context of parents of children with cancer. Thus, these items are very useful for detecting individuals who would benefit from specific psychological interventions. Regarding the difficulty, it should be noted that the ordered response category “rarely” was chosen the least frequently and was irrelevant for items 1, 2, 3, 4, 6, 8, and 9.

Regarding the third aim of estimating the information functions of the nine items, there was a difference in the amount of information provided by the items. The items that provided more information on positive mental health were numbers 4 (“In general, I am confident”), 8 (“Much of what I do brings me joy”), and 3 (“All in all, I am satisfied with my life”), while the item that provided the least information was number 1 (“I am often carefree and in good spirits”). Items 2, 5, 6, and 7 provided an intermediate amount of information.

Regarding the fourth aim, estimating the scores in the latent trait, the findings obtained indicate that the information function had a negative asymmetric shape, with greater precision for scores in the left half of the distribution than those in the right half. Consequently, the scale is more accurate for measuring low levels of positive mental health than high levels. In turn, the profiles are bimodal, exhibiting two high-precision peaks in mean values. Overall, professionals using the PMHS with parents of cancer patients can be confident that its measurements are reliable if the caregiver has a low or medium level of positive mental health, without the need for further analysis. Specifically, the objective of the scale is to detect persons (parents) who may require help due to poor positive mental health [19].

The fifth aim of the study concerned the convergent and divergent validity of the PMHS. The expectation was that there would be significant relationships of PMHS scores with the validation criteria; namely, positive associations with resilience, well-being, and quality of life, and negative associations with depression, anxiety, and caregiver burden. The data did not support these hypotheses, revealing that PMHS scores were independent to five of the six constructs and weakly associated with well-being. Although non-normality of distribution is not necessarily a problem in the data, since it is more common in applied research [63,64], it may not be the optimal condition for exploring construct validity, given its effects on type I and/or type II errors [65,66]. Because of this non-normality of data, the correlations were additionally examined using Spearman’s correlation analyses; these analyses yielded the same results as Pearson’s product–moment correlation analyses. Furthermore, the scatterplots did not show nonlinear relationships. In each of them, a cloud of points with a quadrangular shape appeared, which is typical of two independent variables. It is also essential to consider that the instruments used to assess the validity of the PMHS did not show good fit indices in the CFAs performed on the present sample; therefore, the correlation with their scores could be misleading. If we consider these results valid, positive mental health in this study was independent of resilience, depression, anxiety, caregiver burden, and quality of life. It is a very different concept from those previously studied in psychology, as the authors of the scale remarked [19]. Perhaps the PMHS measures the ability to overcome adversity by maintaining a positive and active attitude without giving up.

Some relevant insights from the clinical point of view can be obtained by looking at the distribution of positive mental health in the present sample of primary caregivers. The negative bias is striking, which makes it easy to identify individuals with very poor mental health. Those individuals who score lower could be those who would benefit more from an intervention. If we consider that the items have high discrimination, reapplying the instrument after an intervention would make it possible to adequately measure whether these caregivers have improved in their positive mental health. On the other hand, the test information function could also provide relevant clinical information. This function indicates that the PMHS provides more information on low levels of positive mental health and very little information on high levels of this construct, which could suggest that the instrument requires higher indicators (items) of positive mental health. This also suggests that this instrument applied alone might not be enough to identify caregivers with greater mental health; therefore, clinicians would benefit from measuring other related constructs; for example, resilience or quality of life.

Regarding the limitations of this study, the sampling was not probabilistic; thus, the estimates should be interpreted with caution, even within the population from which the sample was drawn (parents of children undergoing cancer treatment in the Hospital Infantil de México Federico Gómez, National Institute of Health, in Mexico City) and especially considering that the evaluated parents expressed low levels of the construct. A related problem is the imbalance in the sex ratio, which is an important limitation for the generalization of the results of the present research. If the evaluated sample contains few male participants, the statistical models will have much uncertainty (greater error) in the measurement of the construct. Another limitation is the use of only self-report measures. Future validity studies should include behavioral and physiological measures. Another limitation is that convergent and divergent validity must be explored with other measures of association that are sensitive to the different types of validity; on the other hand, it may also be necessary to estimate correlations between latent scores or scores derived directly from IRT or structural equation modeling (SEM). These latent scores control for measurement error; correspondingly, correlations tend to be higher, which emphasizes the need to use the methods implemented in the present study. Another limitation is that the scale lacks information on high levels of positive mental health. The items were not able to measure high levels. Similarly, parents with lower-than-average trait scores were more likely to select the *Always* response. It is likely that more items will be needed to explore the highest levels of this construct.

## 5. Conclusions

IRT-based analysis provided additional information to classical test theory and allowed the PMHS to be validated in the present study. Regarding the research questions (implicit in the five specific aims), the IRT GRM had a better fit than the GPCM and validated the single latent trait model. The nine items were discriminative. The *Rarely* response was irrelevant for seven of the nine items. The information provided by the items was not homogeneous: items 3, 4 and 8 were the most informative, and item 1 was the least informative. The latent trait estimate was more accurate for low levels of mental health than for high levels; however, its maximum precision was achieved at medium levels. The scale was independent of caregiver depression, anxiety, burden, quality of life and resilience. Higher PMHS scores were associated with low subjective well-being. Thus, positive mental health may represent a way to overcome adversity by maintaining a positive and active attitude without giving up. We suggest that this hypothesis should be examined in future studies.

The use of the one-factor model for the PMHS is recommended for parents of children with cancer. In future studies, the use of probability sampling and an IRT perspective is recommended. In the present study, stability was not estimated; thus, future studies should estimate scores at least at two timepoints. The invariance of parameters and intercepts between groups was not tested since the eligible samples were imbalanced. Therefore, the evaluation of invariance (according to sex of the caregiver and cancer patient, etc.) based on nonproportional stratified sampling (with equiprobable or balanced strata) is recommended. This type of contrast will help establish the invariance of the estimated psychometric parameters or elucidate the differences, as performed previously in the German population [19]. It is very important to continue exploring the construct validity of the PMHS to establish what it truly measures. Once this information has been determined, the scale will be eligible for widespread use and can be disseminated through a website that facilitates its application and interpretation, similar to other instruments [67]. Primary caregivers of children with cancer are a population at risk of multiple problems, both physical and psychological, including financial problems; therefore, having a tool such as the PMHS is highly relevant to identify caregivers who suffer from diminished positive mental health. These caregivers are the ones who would benefit the most from an intervention, which would directly impact their quality of life and indirectly that of the children they care for.

## Figures and Tables

**Table 1:** Summary statistics of sociodemographic variables.

Sociodemographic Variable		*n*, Mean	%, SD
Sex			
	Male	116	18.6
	Female	507	81.4
Schooling			
	No schooling	17	2.7
	Primary school	123	19.7
	Middle school	278	44.6
	High school	159	25.5
	University or college	46	7.4
Occupation			
	Homemaker	405	65
	White-collar worker	86	13.8
	Merchant	58	9.3
	Blue-collar worker	26	4.2
	Unemployed	48	7.6
Marital status			
	Married	252	40.4
	Living together	241	38.7
	Separated	51	8.2
	Single	53	8.5
	Divorced	18	2.9
	Widowed	6	0.9
	Other	2	0.3
Income per month			
	<141 USD	385	61.8
	141–281 USD	137	22.0
	282–563 USD	83	13.3
	>563 USD	18	2.9
Religious affiliation			
	Catholic Christian	502	80.6
	Non-Catholic Christian	75	12
	No religion	46	7.4
Age (years)		31.7	7.55
Number of children		2.32	1.17

Note: *n* = frequency, % = percentage, mean = arithmetic mean, SD = standard deviation.

**Table 2:** Fit comparison between the GPCM and GRM.

Model	AIC	AICc	SABIC	HQ	BIC	logLik	χ^2^
GPCM	10,599.28	10,606.46	10,655.97	10,676.83	10,798.84	−5254.64	134.6
GRM	10,474.95	10,482.12	10,531.64	10,552.50	10,674.50	−5192.47	124.33

Note: GPCM = generalized partial credit model, GRM = graduated response model, AIC = Akaike information criterion, AICc = sample size-adjusted AIC, BIC = Bayesian information criterion, HQ = Hannan-Quinn criterion, SABIC = sample size-adjusted BIC, logLik = likelihood ratio test, χ^2^ = chi-square test.

**Table 3:** GRM discrimination and difficulty parameter estimates for PMHS.

Number	Item	*a*	*b*_1_	*b*_2_	*b*_3_	*b*_4_
1	I am often carefree and in good spirits.	0.64	−4.17	−3.69	−0.86	1.92
2	I enjoy my life.	1.9	−2.41	−2.09	−0.99	−0.14
3	All in all, I am satisfied with my life.	2.81	−2.24	−1.77	−1.02	−0.48
4	In general, I am confident.	3.41	−2.37	−1.95	−1.22	−0.67
5	I manage well to fulfill my needs.	2.21	−3.18	−2.8	−1.48	−0.7
6	I am in good physical and emotional condition.	1.48	−2.62	−1.98	−0.55	0.23
7	I feel that I am actually well equipped to deal with life and its difficulties.	2	−3.05	−2.69	−1.18	−0.46
8	Much of what I do brings me joy.	3.02	−2.69	−2.22	−1.07	−0.27
9	I am a calm, balanced human being.	2.52	−2.44	−2.04	−0.9	−0.08

Note: *a* = discrimination parameter from the GRM model, *b_1_* = difficulty threshold for category two or more (i.e., *Rarely* or higher), *b_2_* = difficulty threshold for category three or more, *b_3_* = difficulty threshold for category four or more, *b_4_* = difficulty threshold for category five.

**Table 4:** Correlations with the PMHS.

Measures of Association	RESI-M	BDI-II	BAI	ZBI	WHOQoL	WHO-WBI
Linear	−0.044	0.058	0.065	0.030	−0.002	−0.105 **
Monotonic	−0.050	0.055	0.072	0.022	−0.025	−0.109 **
Descriptive information						
M	130.77	13.79	14.78	23.24	83.63	19.23
SD	16.04	9.84	13.47	12.27	11.09	5.77
Skew	0.102	1.029	1.239	1.133	0.070	−0.170
Kurtosis	0.179	1.370	0.956	2.941	−0.222	−0.601

Note: RESI-M = Measurement Scale of Resilience in Mexicans, BDI-II = Beck Depression Inventory, second edition. BAI = Beck Anxiety Inventory, ZBI = Zarit Burden Interview, WHOQoL = World Health Organization Quality of Life–short version, WHO-WBI = World Health Organization Well-Being Index. Linear: Pearson’s product-moment correlation coefficient. Monotonic: Spearman’s rank correlation coefficient. *p* values from a two-tailed test: ** *p* < 0.01. Descriptive statistics: M = arithmetic mean, SD = sample standard deviation, skew = Fisher’s coefficient of skewness g_1_ based on the sample-standardized third cumulant, kurtosis = Fisher’s coefficient of kurtosis g_2_ based on the sample-standardized fourth cumulant.

## Data Availability

The raw data supporting the conclusions of this article will be made available by the authors without undue reservation.
